# Arbuscular Mycorrhizal Fungi Alleviate Low Phosphorus Stress in Maize Genotypes with Contrasting Root Systems

**DOI:** 10.3390/plants11223105

**Published:** 2022-11-15

**Authors:** Liyan Liang, Baoxing Liu, Di Huang, Qiqiang Kuang, Tingting An, Shuo Liu, Runjin Liu, Bingcheng Xu, Suiqi Zhang, Xiping Deng, Andrew Macrae, Yinglong Chen

**Affiliations:** 1 State Key Laboratory of Soil Erosion and Dryland Farming on the Loess Plateau, Northwest A&F University, Xianyang 712100, China; 2 College of Forestry, Northwest A&F University, Xianyang 712100, China; 3 College of Natural Resources and Environment, Northwest A&F University, Xianyang 712100, China; 4 Institute of Mycorrhizal Biotechnology, Qingdao Agricultural University, Qingdao 266109, China; 5 Programa Pós-Graduação de Biotecnologia Vegetal e Bioprocessos da Universidade Federal do Rio de Janeiro, Av. Prof. Rodolpho Paulo Rocco, s/n-Prédio do CCS-Bloco K, 2° Andar-Sala 032, Rio de Janeiro 21941-902, Brazil; 6 Instituto de Microbiologia Paulo de Góes da Universidade Federal do Rio de Janeiro, Av. Prof. Rodolpho Paulo Rocco, s/n-Prédio do CCS-Bloco I, 1° Andar-Sala 047, Rio de Janeiro 21941-902, Brazil; 7 The UWA Institute of Agriculture, School of Agriculture and Environment, The University of Western Australia, Perth 6009, Australia

**Keywords:** maize, root system architecture, AMF, P efficiency, low P stress

## Abstract

Soil available phosphorus (P) is one of the main factors limiting plant growth and yield. This study aimed to determine the role of arbuscular mycorrhizal fungi (AMF) in P-use efficiency in two maize genotypes with contrasting root systems in response to low P stress. Maize genotypes small-rooted Shengrui 999 and large-rooted Zhongke 11 were grown in rhizoboxes that were inoculated with or without AMF (*Funneliformis mosseae*) under low P (no added P) or optimal P (200 mg kg^−1^) for 53 days. Low P stress significantly inhibited shoot and root growth, photosynthesis, tissue P content, and root P concentration in both genotypes. Shengrui 999 was more tolerant to P stress with less reduction of these traits compared to Zhongke 11. Shengrui 999 had a higher AMF infection rate than Zhongke 11 at both P levels. Under P deficit, inoculation with AMF significantly promoted plant growth and P uptake in both genotypes with more profound effects seen in Zhongke 11, whilst Shengrui 999 was more dependent on AMF under optimal P. Low P stress inhibited the growth and physiological attributes of both genotypes. The small-rooted Shengrui 999 was more tolerant to low P than Zhongke 11. Inoculation with AMF alleviates low P stress in both genotypes with a more profound effect on Zhongke 11 at low P and on Shengrui 999 at high P conditions.

## 1. Introduction

Phosphorus (P) is one of the key nutrients that is necessary for plant growth and development, and it is mainly absorbed and utilized by plant roots via the phosphate transporter protein carriers. However, most of the P in the soil exists in the form of insoluble organic P, which is also characterized by high adsorption and low mobility, leading to the available P being scarce in soil [1]. The scarcity of available P in soils can cause slow plant growth, delayed flowering, low fruit set, and eventually yield reduction [2,3]. Maize (*Zea mays* L.), as an important grain crop, feed crop, industrial material, and energy resource. It is one of the most widely planted crops in the world with the highest total production [4,5]. However, P deficiency in the soil is one of the major factors limiting maize growth and productivity [6,7,8].

Large amounts of P fertilizer have been widely applied to meet the plants requirement for P ensuring plant growth and to achieve a high yield in maize production. However, the excessive use of chemical fertilizer not only inhibits soil beneficial microorganisms but also causes environmental pollution [9,10]. Plants have evolved a range of strategies in response to low P stress [11,12], such as alteration in root morphology, the release of root exudates [13,14], forming arbuscular mycorrhizal (AM) associations [15,16,17], and cluster roots [6,18]. AM fungi can sense low P concentrations in soils with high nutrient uptake rates and P transportation rates. In response to this, the external mycelium increase the area of P uptake by plants. AMF can influence root morphology and change the distribution of roots in the soil to adapt to the low P stress [19,20].

The aim of this study was to investigate how mycorrhizal inoculation and root morphological traits respond to low P stress in two maize genotypes with contrasting root systems elected from recent studies using a semi-hydroponic root phenotyping system [21]. The study intended to test the following hypotheses: (1) different maize genotypes differ in their tolerance to low P stress, (2) AM fungal inoculation alleviates maize plants to low P stress, and (3) genotypic differences exist in the mitigation of low P stress by AM fungal inoculation.

## 2. Results

### 2.1. Effect of Low P Supply on Plant Growth and P Efficiency

**Figure 1:**
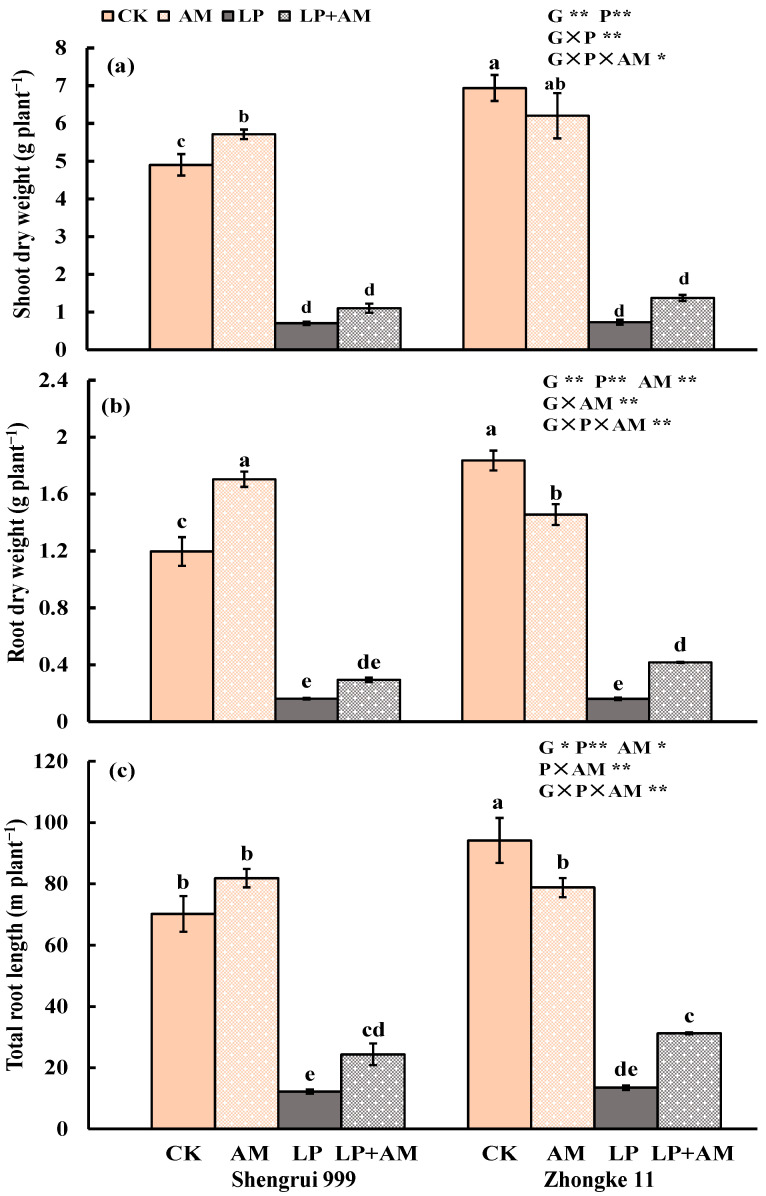
Shoot dry weight (g plant^−1^) (**a**), root dry weight (g plant^−1^) (**b**), and the total root length (m plant^−1^) (**c**) of two maize genotypes (Shengrui 999 and Zhongke 11) under four treatments. Note: CK (optimal P, 200 mg kg^−1^), AM (optimal P + arbuscular mycorrhizal fungal inoculation), LP (low P, no added P), and LP + AM (low P + arbuscular mycorrhizal inoculation). For each trait, bars with a different letter indicate significant difference between treatments and genotypes (*p* > 0.05); data were mean ± SE (n = 4). ANOVA results of three factors (G, genotype; P, phosphorus; AM, arbuscular mycorrhiza) and their interactions are presented for each treatment (*, *p* ≤ 0.05; **, *p* ≤ 0.01). This note applies to other figures when appropriate.

Low P stress exerted a significant inhibitory effect on shoot height, shoot dry weight, and root dry weight (all *p* < 0.01), but had no significant effect on root–shoot dry mass ratio of both maize genotypes regardless of inoculation (Table 1, Figure 1a,b). Compared to optimal P (CK), shoot height was reduced by 39.0%, and the shoot dry weight and root dry weight were 7.27 times and 8.50 times lower under low P conditions, respectively. (Table 1, Figure 1a,b).

Root morphological traits also had significant responses to low P stress. When compared to the CK treatment, root diameter significantly decreased by 6.90% (Table 1). The reduction in the total root length, root surface area, and root volume for the two maize genotypes was 5.42, 5.69, and 6.67 times lower, respectively, than the CK treatment (all *p* < 0.01) (Figure 1c, Table 1). 

**Figure 2:**
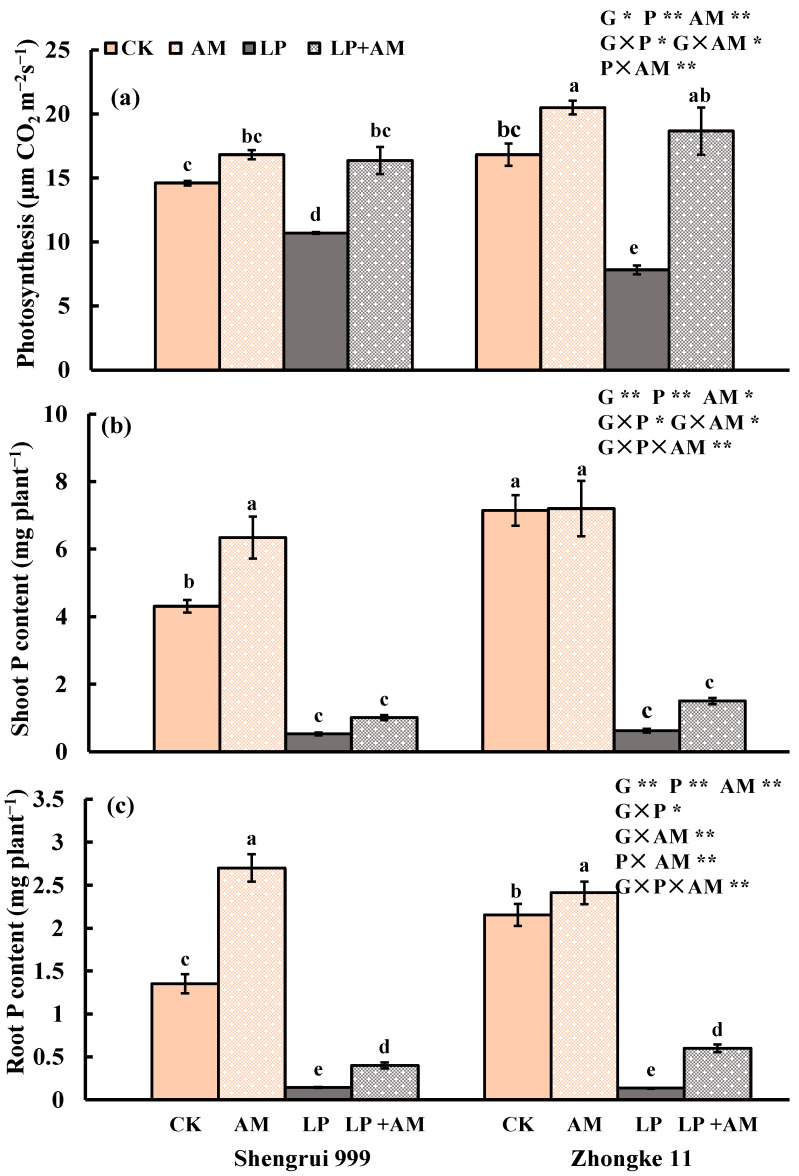
Photosynthesis (μm CO_2_m^−2^s^−1^) (**a**), shoot P content (mg plant^−1^) (**b**), and root P content (mg plant^−1^) (**c**) of two maize genotypes (Shengrui 999 and Zhongke 11) under four treatments (*, *p* ≤ 0.05; **, *p* ≤ 0.01).

Low P stress significantly reduced the photosynthesis rate by 21.9%, the decreased amount of shoot P content and root P content were 8.97 times and 11.53 times lower than that of the shoot P content and root P content under high P conditions, respectively (Figure 2). P acquisition efficiency, P utilization efficiency, and AM infection rate did not differ significantly between the two P levels (*p* > 0.05) (Table 1). 

### 2.2. Genotypic Variation in Response to Low P Stress

Without AMF inoculation, the two genotypes exhibited significant differences in response to low P stress with a greater inhibition on shoot and root growth in Zhongke 11 than in Shengrui 999 (Table 1, Figure 1 and Figure 2). Under optimal P, Zhongke 11 had larger values in shoot-, root-, and P-related traits than Shengrui 999. Compared with CK, LP significantly reduced the shoot height of Shengrui 999 by 32.6% and Zhongke 11 by 45.6%, the root dry weight of Shengrui 999 by 86.7% and Zhongke 11 by 91.3%, the root–shoot dry mass ratio of Shengrui 999 by 0.04% and Zhongke 11 by 0.15%, photosynthesis of Shengrui 999 and Zhongke 11 by 26.7% and 53.4%, respectively, the PAE of Zhongke 11 was 19.6% and no change in Shengrui 999 (Table 1, Figure 1 and Figure 2).

### 2.3. Effect of AMF Inoculation on Plant Growth and P Efficiency

**Figure 3:**
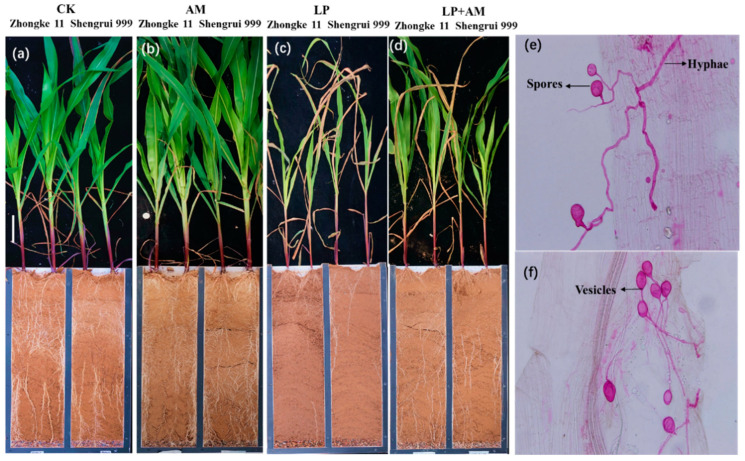
Example plant photos (**a–d**) and microscopy images of AM structure (spores, hyphae, vesicles, and arbuscules) stained with acid fuchsin (**e**,**f**) of two maize genotypes (Shengrui 999 and Zhongke 11) under four treatments that were grown in rhizoboxes for 53 days after transplanting. Note: (**a**), CK (optimal P, 200 mg kg^−1^); (**b**), AM (optimal P + arbuscular mycorrhizal fungal inoculation); (**c**), LP (low P, no added P); (**d**), LP + AM (low P + arbuscular mycorrhizal fungal inoculation); (**e**) (Zhongke 11 under LP + AM); (**f**), (Shengrui 999 under AM).

The AMF infection rate of the two maize genotypes was averaged at 33.3% and 35.2% under optimal P and low P (Table 1). The AMF infection rates of Shengrui 999 were higher than Zhongke 11 under both P conditions. There was no significant difference in the AMF infection rate that was caused by phosphorus levels (Table 1). Maize roots that were inoculated with AM fungus have hyphae, spores, and vesicles (Figure 3e,f). Mycorrhizal structures were not observed in the uninoculated maize roots. Uninoculated maize roots for both maize genotypes were significantly reduced, and the shoot growth was significantly decreased after low P stress (Figure 3a,c). AMF inoculation under low P conditions effectively alleviated low P stress (Figure 3c,d). In general, inoculation with AMF significantly increased shoot height, root dry weight, root–shoot dry mass ratio, photosynthesis, root P content, shoot and root P concentrations, PAE and AM infection rate (all *p* < 0.01), and the total root length, root surface area, and shoot P content (all *p* < 0.05), but significantly decreased root diameter (*p* < 0.01) and PUE (*p* < 0.05) of both genotypes (Table 1, Figure 1 and Figure 2). There were significant interactions between G, P, and AM treatments in nine traits, for example, shoot and root dry weight, total root length, and the shoot and root P content (Figure 1 and Figure 2).

### 2.4. Alleviative Effect of AMF Inoculation on Plant Growth, Photosynthesis, and P Efficiency under Low P Stress

Inoculation of AMF significantly affected all 15 traits of both maize genotypes (Table 2). Under low P conditions, AMF treatment significantly increased the shoot height by 9.73%, shoot dry weight by 72.7%, root dry weight by 1.22 times, and root–shoot dry mass ratio by 28.3% of both genotypes, compared to non-mycorrhizal treatment (Table 2). AM inoculation also significantly affected root morphological traits: total root length, root surface area, and root volume significantly increased by 1.17 times, 1.11 times, 98.5%, respectively, but significantly decreased root diameter by 7.14% of both genotypes (Table 2). AM inoculation significantly promoted photosynthesis by 89.4%, PAE, shoot P content, root P content by 35.0%, 1.18 times, 2.57 times, respectively, but significantly inhibited PUE by 27.5% (Table 2).

### 2.5. Genotypic Variation in Response to AMF Inoculation

Under the optimal P condition, Shengrui 999 responded more to AMF than Zhongke 11 among the 15 measured traits. Shengrui 999 showed a positive response to AMF inoculation treatment in 10 traits (shoot height, shoot dry weight, root dry weight, root–shoot dry mass ratio, root surface area, shoot and root P contents, shoot and root P concentrations, and PAE) (Table 1, Figure 1 and Figure 2), had a negative response in two traits (root diameter and PUE) and no significant response in the three other traits (total root length, root volume, and photosynthesis) (Table 1, Figure 1 and Figure 2). However, Zhongke 11 positively responded to AMF inoculation in four traits (shoot height, photosynthesis, and root P concentration and content), no significant response in six traits (shoot dry weight, root-to-shoot dry mass ratio, shoot P content, shoot P concentration, PAE, and PUE), and negative response in five traits (root dry weight, root diameter, total root length, root surface area, and root volume) (Table 1, Figure 1 and Figure 2)

Under low P conditions, Zhongke 11 was more responsive to AMF than Shengrui 999. Inoculation with AMF significantly increased the values of seven traits (shoot height, total root length, photosynthesis, shoot and root P concentration, root P content, and PAE) for both genotypes (Table 1, Figure 1 and Figure 2). Zhongke 11 had significant positive responses in root dry weight, root–shoot dry mass ratio, and root surface area, but Shengrui 999 had no significant response in these traits (Table 1, Figure 1 and Figure 2). Inoculation only significantly inhibited the root diameter of Shengrui 999 but had no remarkable change on Zhongke 11 (Table 1).

### 2.6. Accumulated Root Length

**Figure 4:**
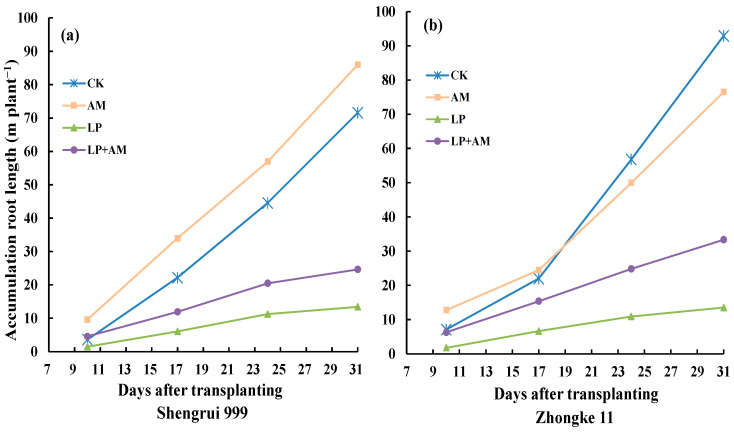
Accumulated visible root length (m plant^−1^) of Shengrui 999 (**a**) and Zhongke 11 (**b**) via the transparent wall of the rhizobox on 10, 17, 24, and 31 days after transplanting (DAT) under four treatments 2.7 Correlations among morphological and physiological traits.

Visual root growth through the glass plates showed increased differences among the four treatments with time in both genotypes (Figure 4). Low P treatments (LP and LP + AM) significantly inhibited root development observed on 17, 24, and 31 DAT regardless of AMF inoculation in both genotypes as evidenced in accumulative root length. Shengrui 999 had remarkable responses to AMF inoculation in root growth compared to non-inoculation treatments under respective P levels (Figure 4a), while Zhongke 11 only showed significant positive mycorrhizal response in root growth under low P conditions and had a negative mycorrhizal response under the optimal P condition (Figure 4b). Such genotypic variations in response to AMF inoculation in accumulated root length at the early growth stages up to 31 DAT was mirrored by shoot dry weight, total root length, and root dry weight of both genotypes at the final harvest on 53 DAT (Figure 1).

**Figure 5:**
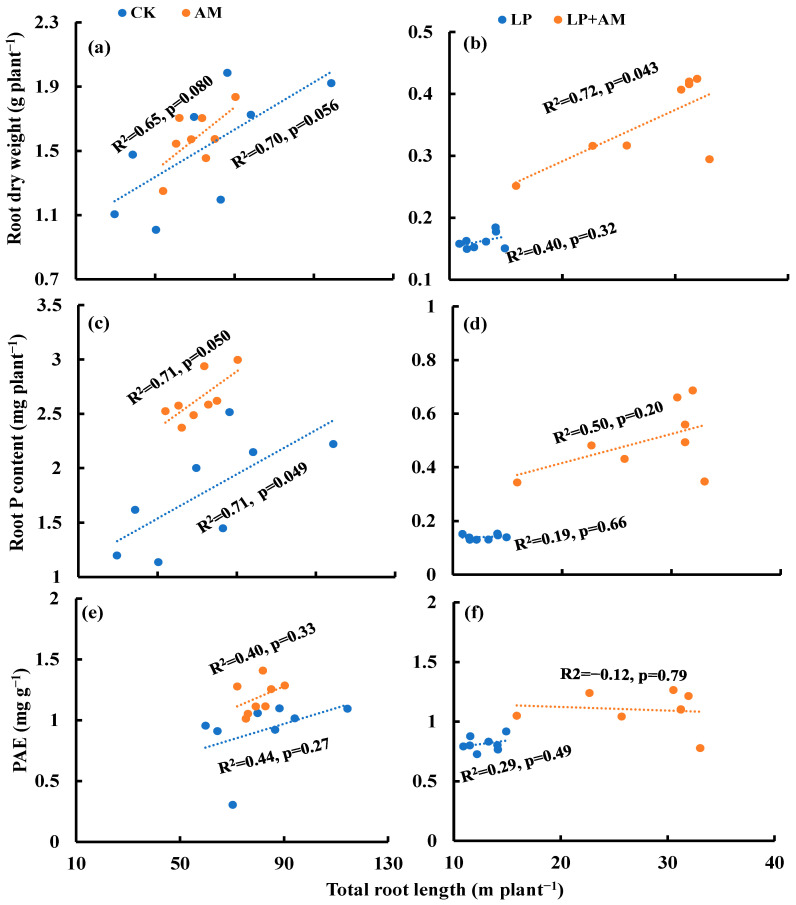
The relationship between total root length and root dry weight (**a**,**b**), root P content (**c**,**d**), and PAE (**e**,**f**) under four treatments. Note: Correlation coefficient of each trait from Pearson correlation analysis, data from each maize plant. PAE, phosphorus acquisition efficiency.

Under optimal P, AMF inoculation diminished the correlation between the total root length and root dry weight (Figure 5a), and the correlation between the total root length and PAE (Figure 5e) when compared to non-inoculation treatment. Under the low P condition, AMF inoculation enhanced the correlation between the total root length and root dry weight (Figure 5b), total root length and root P content (Figure 5d), and also diminished the correlation between the total root length and PAE (Figure 5f).

**Figure 6:**
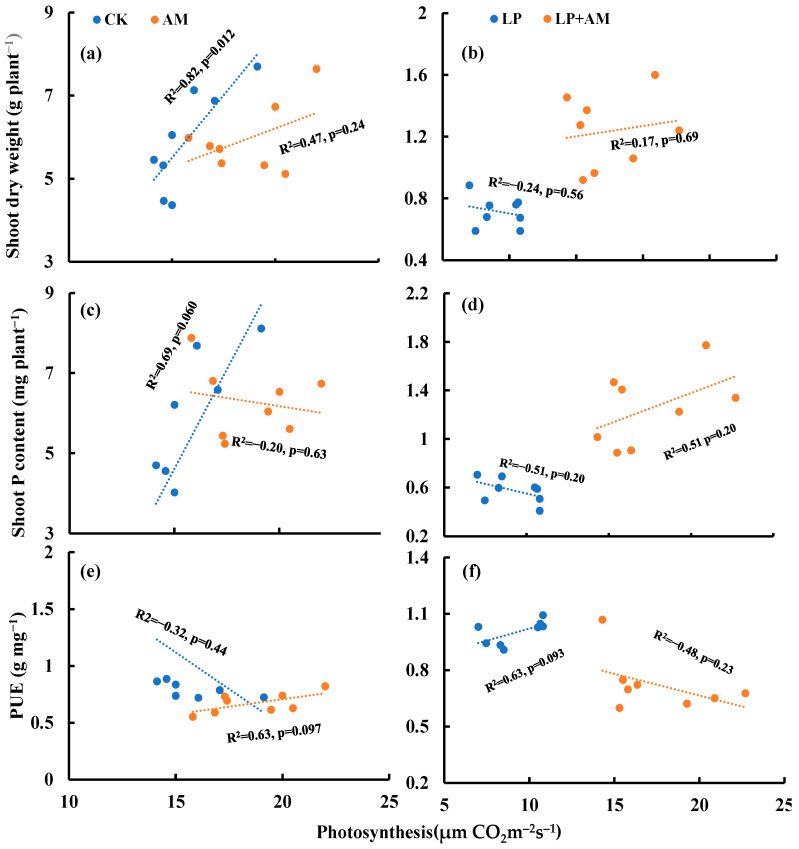
The relationship between photosynthesis and shoot dry weight (**a**,**b**), shoot P content (**c**,**d**), and PUE (**e**,**f**) under four treatments. Note: Correlation coefficient of each trait from Pearson correlation analysis, data from each maize plant. PUE, phosphorus utilization efficiency.

Under optimal P, AMF inoculation decreased the correlation between photosynthesis and the shoot dry weight (Figure 6a) and shoot P content (Figure 6c) but increased the positive correlation between photosynthesis and PUE (Figure 6e). Under low P stress, AMF inoculation decreased the correlation between photosynthesis and shoot dry weight (Figure 6b), photosynthesis, and PUE (Figure 6f).

## 3. Discussion

### 3.1. Root Morphological Traits in Response to Low P Stress

**Figure 7:**
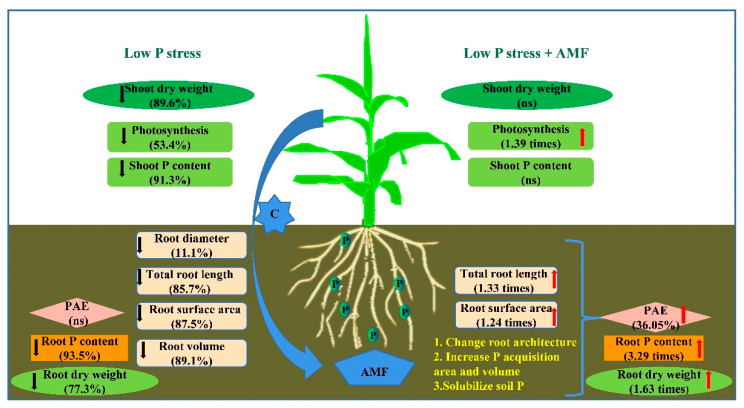
The role of arbuscular mycorrhizal (AM) fungi in improving plant growth, physiology, and alleviation of low P stress in maize. Note: Data for each trait under low P stress were compared to optimal P treatment (CK). Data for each trait under low P stress + AMF were compared to low P treatment (LP) on the respective trait of genotype Zhongke 11 as an example, respectively. Arrows indicate significant increase (in red) or decrease (in black) ns, non-significant effect.

The plants root system architecture (RSA) traits include three main categories: topological properties, geometric properties, and physiological properties [22]. RSA is determined by genetics and environmental factors, and both play a direct key role in the absorption of P. Root architecture characteristics affect their P uptake, and in turn, P scarcity in the plant growing environment also affects the plant root architecture. This study observed alterations in the root morphological traits (total root length, root diameter, root surface area, and root volume) of both maize genotypes. Plants tolerate to low P stress by making their roots thinner (Figure 7). Fine roots and fine root structures contribute to exploited small, localized soil volumes with high efficiency and increase plant PAE under low P availability [23,24]. Plants have evolved some strategies including adjustment of P absorption through modifying RSA traits to ensure their survival, growth, and development [25,26,27]. Genotypes that are efficient in P acquisition in low P soils develop adaptive RSA traits such as increased root length and root-hair density and length, and optimal placement of roots in soil [25,28]. When soil P is scarce, many plants inhibit primary root elongation and stimulate lateral root growth to build shallow root systems that concentrate in the topsoil due to soil P decreasing with increasing soil depth [29,30,31,32]. For maize, most crown roots are distributed in the topsoil which supports the above view [33,34]. Molecular studies have shown that low P stress stimulated the overexpression of RSA-related genes with more profound expression in maize genotypes with higher P efficiency [35]. A genome-wide association mapping study identified novel genes and loci that were associated with root morphological traits and P acquisition and use efficiency in chickpea [36]. PIP5K genes regulating root hair elongation in response to P deficiency have been reported in Arabidopsis [37], and SIZ1 and AtSIZ1 regulate RSA by inhibiting primary root elongation and promoting lateral root formation and root hair development [38], while OsPHR2 genes regulate rice root hair growth and root elongation [39]. Exploring the relationship between RSA and P acquisition and P efficiency can provide some theoretical support for the screening and breeding of P-efficient genotypes.

### 3.2. The Role of AMF in Plant Alleviation under Low P Stress

It is well accepted that inoculation with AMF improves P acquisition, especially when the availability of soil P is low. The extending hyphae of AMF can greatly increase the absorption area and can help solubilize P in the soil, thus enhancing P uptake and promoting plant growth and yield, particularly for species with poor root development [4,40,41,42,43,44,45]. This study also demonstrated the alleviation role of AMF under low P stress by significantly increased maize P uptake and improved shoot and root growth.

The formation of AM can alter RSA to tolerate low P stress, and the effect of AM on RSA is usually attributed to the improvement of P absorption. In addition, plants themselves can resist low P stress by reducing the diameter of the root system, making the roots thinner, and producing more branches. This study showed that among the morphological traits, average root diameter was significantly reduced after inoculation of AMF. This result may indicate possible complementarity between root morphology and mycorrhiza in P uptake under low P stress. Ma et al., (2018) suggested that plants evolved thinner roots to reduce their dependence on AMF for nutrient uptake [46] (Figure 7). The results of Wen et al., (2019) of the response of 16 crops to low P stress in terms of root morphology, root secretion, and inoculum showed that species with thinner roots showed a stronger response in root branching, first-order root length, and specific root length of the whole root system, Conversely, species with thicker roots exhibited higher colonization by AMF and/or more P-mobilizing exudates in the rhizosheath [47]. The results of [48,49,50] also indicated some complementarity between root morphology and mycorrhiza for phosphorus absorption under plant low P stress.

AMF inoculation also significantly increased photosynthesis of both maize genotypes. The improvement in photosynthesis that was associated with AMF is mainly due to the improvements in the availability of P and other nutrients that supply the photosystem [51]. The carbon sink of AMF to increase photosynthetic rates is a compensatory mechanism safeguarding carbon supply for plant development [52]. The results on the physiology of maize leaves under low P stress and inoculation of AMF also showed that low P stress significantly reduced photosynthesis and respiration in maize leaves, but the increase in phosphorus nutrient uptake after inoculation with AMF affected gas exchange, photosynthetic enzymes, and chlorophyll in maize leaves, ultimately alleviating the negative effects of low phosphorus stress on photosynthesis and respiration [53] (Figure 7). Rezacova et al., (2018) studies on typical C3 and C4 plants also reported that the stimulation of carbon sink redistribution and photosynthesis was upregulated after the establishment of AMF under low P stress [54].

These results indicate that AMF inoculation can alleviate low P stress through the improvement of RSA, plant growth, photosynthesis, and P uptake (Figure 7). Most AMF-inoculated plants use two major pathways for the uptake of nutrients: direct uptake via the root epidermis, including root hairs, and indirectly through fungal structures (arbuscules) made by AMF. Tracing the relative contribution of direct pathway and AM pathway by physiologically labeled Pi demonstrated that the contribution of the AM pathway to plant P uptake varies from a small fraction to almost all in plants [16,55]. The AM pathway greatly reduces the dependence of the plant on the direct uptake pathway. The plant PHT1 P transporter family exhibited a role that was specific to both pathways [55,56]. The molecular analysis results supported the interaction between the AM pathway and the direct phosphorus absorption pathway. Studies on potato, rice, and maize found that the PHT1 P transporter that is expressed in cortical cells induced by P starvation was down-regulated in mycorrhiza which may due to phosphorus state in plants [57,58,59]. It was found that AMF inoculation increased the expression of ZmPht1;6 in maize [60,61]. The abundance of AMF external hyphae and the accumulation of transcripts encoding PHT1 phosphate transporters had a strong correlation with PAE in maize plants following inoculation [62].

### 3.3. Genotypic Variations in Response to Low P Stress and AMF

The two genotypes showed significant genotypic variation in RSA including total root length, total surface area, and root volume, confirming our previous observations from phenotyping studies [21]. The large-rooted Zhongke 11 had greater shoot dry weight, plant P acquisition, and PAE than the small-rooted Shengrui 999 when soil P was sufficient. Gu et al., (2016a) observed the associations between P efficiency and root system size in maize [31]. Many studies support that genotypes with larger root systems often result in higher P absorption efficiency, enabling plants to obtain more P from the soil and thereby affecting plant growth, photosynthesis, and yield [50,63,64,65]. Maize genotypes with a larger root–shoot ratio, more lateral root branches, higher root hair density, and longer root hair length were superior in P uptake compared to genotypes with opposite root traits [66,67,68]. These phenomena were also reported in rapeseed (*Brassica napus*), in which the P-efficient genotype developed more dominant root morphological traits, intensive rhizosphere acidification, greater exudation of carboxylates, P uptake, and shoot and root dry weight than a P-inefficient genotype [69]. It has also been reported that there is wide genotypic variation in the root architecture and P efficiency among plant species and genotypes [70,71,72] (Figure 7). The species or genotypic capacity to acquire P is closely related to the differences in root morphological traits. The present study indicated that low P stress had a greater effect on the large-rooted Zhongke11 (P-sensitive) than on the small-rooted Shengrui 999 (also P-tolerant). Apart from RSA, root physiology, in particular rhizosphere carboxylates, has significant effects on P acquisition and efficiency [73,74,75]. Yang et al., (2022) reported that P-efficient maize genotypes have a high capacity to remobilize lipid P and nucleic acid P and promote the shikimate pathway than P-inefficient genotypes [76]. 

This study showed a higher mycorrhizal infection rate in Shengrui 999 (small-rooted) than in Zhongke 11 (large-rooted) (Table 1). Tawaraya (2003) also observed that plant species or genotypes with large and complex root structures have less mycorrhizal colonization than those with simple root structures [77]. However, Kaeppler et al., (2000) showed no significant correlation between mycorrhizal infestation rate and mycorrhizal effects in maize lines under low P stress [78]. The mycorrhizal effect generally depends on: the nutrient conditions of their growth environment, source AMF, plant species or genotypes with contrasting RSA traits, and plant P efficiency [19,79,80,81]. The mycorrhizal effect tends to be positive when the plant is in a nutrient-deprived environment [82,83,84]. The opposite may have a weak or even negative effect on plant growth [85]. The present study demonstrated that the mycorrhizal effect was influenced by soil P status and showed genotypic dependency. Kaeppler et al., (2000) studied maize in low phosphorus stress with AMF and showed no significant mycorrhizal effect compared with a low P-tolerant genotype that was screened under low P conditions without AMF [78]. Hetrick et al., (1992) concluded that the mycorrhizal effect of plants depends to a large extent on their own growth potential under low P conditions without AMF [86,87]. 

## 4. Materials and Methods

### 4.1. Experimental Design, Plant Materials, and AM Inoculum

The experiment was conducted in rhizoboxes in a temperature—controlled glasshouse with day/night temperatures of 23 (±5) °C/16 (±3) °C and an average relative humidity of 65–75% at Northwest A&F University, Yangling (34°16′ N, 108°4″E) from November to January 2020. This study used a randomized complete block design that comprised of two genotypes of maize (*Zea mays* L.), two P treatments, and two arbuscular mycorrhizal (AM) inoculation levels. Genotype Shengrui 999 had a smaller root system while Zhongke 11 had a larger root system and were selected from the recent phenotyping study for root traits [21] and the screening experiment for genotypic variation in P efficiency (Liang et al., under internal review). The P treatments were low P (no added P, soil contained 2.54 mg kg^−1^) and optimal P (200 mg kg^−1^; also referred as “high P” in relation to “low P”). The inoculum that was used in this study was AM fungus *Funneliformis mosseae* BGC NM02A, propagated with *Trifolium repens* using the pot culture method [88]. It comprised of a mixture of sand, spores (approximately 14 spores per gram), mycelia, and infected root fragments. The inoculation treatment comprised of 100 g pot^−1^ (+AM) of the inoculum and the non-inoculation control was given the same dosage of sterilized inoculum with bacterial filter liquor (−AM). There were four replicates in each treatment.

### 4.2. Rhizoboxes, Soil, and Potting

The rhizoboxes (15 cm long, 3 cm wide, 60 cm deep) were constructed from polyvinyl chloride (PVC). One side of the rhizobox was made of transparent plexiglass plate, which can be used to observe the dynamic growth of roots and covered with a black plastic sheet to avoid light exposure to the roots during the experiment. Rhizoboxes were placed on steel stands at an angle of 30° and spaced 0.05 m apart from each another. A total of 32 rhizoboxes (2 × 2 × 2 × 4) were used.

The soil that was used in the experiment was taken from Yangling farmland (0–20 cm soil layer). The soil was air-dried, sieved (2 mm), and mixed with an equal weight of air-dried washed fine river sand. The physical and chemical properties of the mixed soil were as follows: organic matter 3.90 g kg^−1^, total nitrogen 253 mg kg^−1^, available nitrogen 19.6 mg kg^−1^, available P 2.54 mg kg^−1^, available potassium 131 mg kg^−1^, and pH 7.5. For the optimal P treatment (200 mg kg^−1^), P was supplied in the form of Ca(H_2_PO_4_)_2_ and mixed thoroughly with the soil together with the base fertilizer: nitrogen (100 mg kg^−1^) and potassium (120 mg kg^−1^). For the low P treatment, only the base fertilizer was added. 

Each rhizobox was filled with 3.3 kg of the above soil mixture amended with base fertilizer with or without P addition. Prior to the soil filling, the bottom of each rhizobox was covered with a layer (2 cm) of small rocks to allow free draining. Each rhizobox was filled with the soil mixture with the top 10–20 cm layer filled with the soil amended with 100 g of AM inoculum for the AM inoculation treatment. For rhizoboxes without AMF inoculation, 5 mL of the fungicide filtrate of the same amount of AMF inoculum was added to the soil. Finally, the top 2 cm of the rhizoboxes was topped up with the untreated soil mixture. 

### 4.3. Seed Germination, Plant Cultivation, and Maintenance

Uniform seeds were surface sterilized in 10% H_2_O_2_ for 10 min. After rinsing with water, the seeds were soaked in saturated CaSO_4_ solution for 8 h. A total of four homogenous pre-germinated seeds of the same genotype were sown in two holes (two seeds per hole) at a depth of 2 cm in each rhizobox, ensuring that the seeds were in contact with the glass wall. After emergence, two plants from different holes were retained in each rhizobox and were considered as one replication. 

The plants were watered manually from the top as required to maintain the soil water content close to field capacity and the rhizoboxes were randomly repositioned weekly to minimize environmental effects. The root growth was traced once a week commencing on the 10th day after transplanting (DAT). Visible new roots through the glass panels were traced on the transparent sheets using a marker pen. Immediately afterwards, the visible new roots were also marked on the glass panels to facilitate the next observation of the newly grown roots. Root tracing was done 4 times: DATs 10, 17, 24, 31) when some roots had reached the bottom of the rhizoboxes. The traces were scanned at 300 dpi using a portable scanner (Jenkins PS4100, East Bentleigh, VIC, Australia) and root images were analyzed for root length using WinRhizo Pro software (v2009, Regent Instrument, Mobtreal, QC, Canada).

### 4.4. Plant Harvest and Assessments

All the plants were harvested 53 DAT for assessments. Before harvest, the shoot height and leaf number were measured manually and photosynthesis was measured using a portable photosynthesizer at 9:00–11:30 am on a sunny day. The latest fully expanded leaf of each plant was used for measuring photosynthesis. On the day of harvest, plants and root systems in each rhizobox were photographed before and after the glass wall was removed. The two plants in each rhizobox were harvested by cutting the shoots from the roots along the soil layer. The shoots were dried in an air-forced oven at 75 °C for 72 h to a constant weight to determine the shoot dry weights. The root samples were rinsed with tap water, and a small part of roots was randomly cut from multiple directions and stored in 10 mL centrifuge tubes with 70% ethanol solution as mycorrhizal microscopy samples, the rest of the root samples were stored in plastic bags in a 4 °C refrigerator for later root scanning. The roots were scanned in greyscale at 300 dpi using a desktop scanner and were analyzed in WinRHIZO as mentioned above to obtain root morphology data including root length, average root diameter, root surface area, and root volume. After scanning, the roots were oven-dried to determine root dry weights. The root–shoot mass ratio (RSM) was calculated as the root dry weight divided by the shoot dry weight.

The determination of P concentration: the dried root and shoot parts were ground into a powder, then 0.1 g of sample was put into the digestion tube, and 5 mL of concentrated sulfuric acid was added for digestion at 360 °C. The P concentrations of the root and shoot parts were measured by molybdate-blue colori-metric method [89], and P content and efficiency were calculated using following equations [90]:

P contents in shoot (or root) = shoot (or root) dry weight (DW) × P concentration in shoot (or root).

P-acquisition efficiency (PAE) = plant P content/plant dry weight.

P-utilization efficiency (PUE) = shoot dry weight/plant P content

The determination of AMF infection rate: the root samples were cut into 1-cm long pieces, followed by bleaching with 5% KOH (90 C, 20 min), acidifying in 2% HCl (5 min, room temperature), and staining in 0.01% acid fuchsin (overnight, room temperature). The extent of root length colonization was estimated using the gridline intersect method [88]:

AMF infection rate = number of AMF infected root segments/total number of root segments × 100%

### 4.5. Data Analysis

The observed data were calculated and sorted by Microsoft Excel. Values for 16 traits were analyzed by three-way ANOVA using SPSS 20.0 (IBM, Chicago, IL, USA) to test the significance of different factors (genotype, P, and AM) and their interactions (*p* < 0.05, LSD). Pearson’s correlation coefficient was used to analyze the relationship between morphological and physiological traits. The graphs were drawn in Origin 2018 (Microcal, Northampton City, MA, USA).

## 5. Conclusions

The study demonstrated that low P stress significantly inhibited shoot and root growth of both maize genotypes, reduced leaf photosynthesis rate, plant P content, and PAE. Genotypic variation in response to low P stress was observed with a more profound inhibitory effect on the large-rooted genotype Zhongke 11 compared to the small-rooted Shengrui 999. Zhongke 11 was more sensitive to low P stress than Shengrui 999. AMF inoculation significantly promoted plant growth, root–shoot dry mass ratio, photosynthesis rate, P uptake, and PAE under both P conditions. Zhongke11 was more dependent on AMF than Shengrui 999 under low P conditions and *vice versa* when the soil P was optimal. Given that plant available P is generally deficient in soils, the non-renewable nature of P fertilizers, and the high cost and negative environmental impact of heavy fertilizer application, it is crucial to efficiently manage and P applications by planting P-efficient genotypes with AMF inoculation. Future studies with more genotypes and long-term field trials are required to determine the roles of root system architecture, rhizosphere physio-chemistry, and beneficial microbes for maize under low P conditions.

## Figures and Tables

**Table 1:** Treatment effects on shoot height, root surface area, root volume, root diameter, root–shoot dry mass ratio, shoot and root P concentration, PAE, PUE, and AM infection rate of two maize genotypes (Shengrui 999 and Zhongke 11) grown for 53 days in soil-filled rhizoboxes.

Genotype	Treatment	Shoot Height (cm)	Root Surface Area (cm^2^ Plant^−1^)	Root Volume (cm^3^ plant^−1^)	Root Diameter (mm Plant^−1^)	Root to Shoot Dry Mass Ratio	Shoot P Concentration (mg g^−1^)	Root P Concentration (mg g^−1^)	PAE (mg g^−1^)	PUE (g mg^−1^)	AM Infection Rate (%)
Shengrui 999	CK	61.3 b	787 b	9.55 b	0.46 a	0.24 b	0.88 b	1.13 c	0.77 c	1.32 a	0 a
	AM	69.8 a (13.9)	1076 a (36.7)	11.5 a (20.4)	0.41 b (−10.8)	0.30 a (25.0)	1.11 a (25.4)	1.58 a (40.1)	1.21 a (57.1)	0.64 b (−51.5)	33.8 b
	LP	41.3 d (−32.6)	150 d (−80.9)	1.58 c (−83.5)	0.43 b (−6.7)	0.23 b (−0.04)	0.75 b (−15.1)	0.88 d (−22.5)	0.77 c (0)	1.05 a (−20.5)	0 a
	LP + AM	45.3 c (−26.1)	295 c (−62.5)	2.86 c (−70.1)	0.39 c (−14.8)	0.28 ab (0.17)	0.94 ab (6.6)	1.36 b (20.2)	1.03 ab (33.8)	0.79 b (−40.2)	37.4 b
Zhongke 11	CK	69.5 b	1350 a	15.6 a	0.46 a	0.27 ab	1.03 b	1.17 c	1.07 ab	0.74 b	0 a
	AM	71.4 a (2.7)	1024 b (−24.1)	10.8 b (−30.8)	0.41 b (−10.8)	0.24 b (−0.11)	1.16 a (12.3)	1.66 a (41.2)	1.17 a (9.34)	0.70 b (−5.40)	32.7 b
	LP	37.8 d (−45.6)	169 d (−87.5)	1.70 c (−89.1)	0.41 b (−11.1)	0.23 b (−0.15)	0.86 c (−16.7)	0.84 d (−28.5)	0.86 bc (−19.6)	0.95 ab (28.4)	0 a
	LP + AM	41.5 c (−40.3)	379 c (−71.9)	3.65 c (−76.6)	0.39 b (−14.8)	0.31 a (0.15)	1.09 ab (6.2)	1.44 b (22.6)	1.17 a (9.34)	0.66 b (1.08%)	33.0 b
ANOVA	G	**ns**	**	**	**ns**	**ns**	**	**ns**	*	**ns**	**ns**
	P	**	**	**	**	**ns**	**	**	**ns**	**ns**	**ns**
	AM	**	*	**ns**	**	**	**	**	**	*	**
	G × P	**	**	*	**ns**	**ns**	**ns**	**ns**	**ns**	**ns**	**ns**
	G × AM	**	**	**	**ns**	**ns**	**ns**	**ns**	**ns**	**ns**	**ns**
	P × AM	**ns**	**	**	**ns**	**ns**	**ns**	**ns**	**ns**	**ns**	**ns**
	G × P × AM	**	**	**	**ns**	*	**ns**	**ns**	**ns**	**ns**	**ns**

Note: CK = optimal phosphorus (P) (200 mg kg^−1^), LP = low P (no P addition), AM = AMF inoculation under optimal P, LP + AM = AMF inoculation under low P. *, *p* ≤ 0.05; **, *p* ≤ 0.01; ns, non-significant. For each trait, mean data followed by different letters are significantly different among the eight treatments across the two genotypes (*p* ≤ 0.05); data in the brackets are percentages (%) over CK for the same trait indicating a positive effect (increase) or negative effect (decrease). PAE (P acquisition efficiency) = plant P content/plant dry weight; PUE (P utilization efficiency) = shoot dry weight/plant P content. AM infection rate = number of AM fungi infected root segments/total number of root segments × 100.

**Table 2:** Mean values and response (%) of 15 traits of both maize genotypes to arbuscular mycorrhizal (AM) fungus (*Funneliformis mosseae* BGC NM02A) under low phosphorus (LP, no P added) conditions.

Trait	LP	LP + AM	Response (%)	ANOVA
Shoot height (cm)	79.1	86.8	9.73	**
Shoot dry weight (g plant^−1^)	1.43	2.47	72.7	**
Root dry weight (g plant^−1^)	0.32	0.71	122	**
Total root length (m plant^−1^)	25.6	55.5	117	**
Root diameter (mm plant^−1^)	0.84	0.78	−7.14	**
Root surface area (cm^2^ plant^−1^)	319	674	111	**
Root volume (cm^3^ plant^−1^)	3.28	6.51	98.5	**
Root to shoot dry mass ratio	0.46	0.59	28.3	**
Photosynthesis (μm CO_2_m^−2^s^−1^)	18.53	35.1	89.4	**
Shoot P content (mg plant^−1^)	1.15	2.51	118	**
Root P content (mg plant^−1^)	0.28	1	257	**
Shoot P concentration (mg g^−1^)	1.61	2.03	26.1	**
Root P concentration (mg g^−1^)	1.72	2.8	62.8	**
PAE (mg g^−1^)	1.63	2.2	35.0	**
PUE (g mg^−1^)	2	1.45	−27.5	**

Note: **, *p* ≤ 0.01; Responses were positive or negative to AM inoculation compared to non-AM treatment (LP) across two maize genotypes.

## Data Availability

Relevant data that were generated or analyzed during this study are included in this article. Other data are available upon request to the corresponding author.
